# PReS-FINAL-2353: Are rasopathies new monogenic predisposing conditions to the development of systemic lupus erythematosus?

**DOI:** 10.1186/1546-0096-11-S2-P343

**Published:** 2013-12-05

**Authors:** B Bader-Meunier, H cave, N jeremiah, F Rieux-Laucat

**Affiliations:** 1 Hôpital Necker, France; 2 hôpital R.Debré, France; 3 INSERM U768, Paris, France

## Introduction

RASopathies (Noonan syndrome (NS) and Noonan-related syndromes) are neurodevelopmental syndromes resulting from germline mutations in genes that participate in the rat sarcoma/mitogen-activated protein kinases (RAS/MAPK) pathway (*PTPN11, SOS1, RAF, KRAS *or *NRAS *and *SHOC2*). Some monogenic conditions are associated with the development of systemic lupus erythematosus (SLE), and a few reports described the association of SLE, with NS.

## Objectives

We aim to search for a relationship between RASopathy and the development of SLE.

## Methods

We reported for the first time on a 13-year-old boy with NS with loose anagen hair (NSLAH) resulting from mutation in *SHOC2 *who developed an autoimmune disorder which fulfilled four American College or Rheumatology (ACR) criteria for the classification of SLE (polyarthritis, pericarditis, antinuclear antibodies, anti-DNA antibodies). The case report then prompted a literature review by a systematic search for English and French articles on the subjects of RASopathies and SLE that had English abstracts in PubMed from 1966 to 2012.

## Results

We identified seven additional patients with RASopathy and SLE. The male-to-female ratio was 1:1, and age at onset of SLE ranged from 5 to 32 years. The most common features were polyarthritis (7/8 patients), auto-immune cytopenia (4/8 patients) and pericarditis (4/8 patients) while only one patient presented with skin involvement.

## Conclusion

The association of two rare diseases in eight patients suggests that RASopathies may be associated with the development of SLE, which is characterized by a higher male-to-female ratio, a lower rate of skin involvement and a higher rate of pericarditis than "classic" SLE.

## Disclosure of interest

None declared.

